# Human plasma pregnancy-associated miRNAs and their temporal variation within the first trimester of pregnancy

**DOI:** 10.1186/s12958-021-00883-1

**Published:** 2022-01-14

**Authors:** Cécilia Légaré, Andrée-Anne Clément, Véronique Desgagné, Kathrine Thibeault, Frédérique White, Simon-Pierre Guay, Benoit J. Arsenault, Michelle S. Scott, Pierre-Étienne Jacques, Patrice Perron, Renée Guérin, Marie-France Hivert, Luigi Bouchard

**Affiliations:** 1 Department of Biochemistry and Functional Genomics, Faculty of Medicine and Health Sciences (FMHS), Université de Sherbrooke, Sherbrooke, QC Canada; 2 Clinical Department of Laboratory Medicine, Centre Intégré Universitaire de Santé et de Services Sociaux (CIUSSS) du Saguenay–Lac-St-Jean – Hôpital Universitaire de Chicoutimi, Pavillon des Augustines, 305 rue St-Vallier, Saguenay, QC G7H 5H6 Canada; 3 Department of Biology, FMHS, Université de Sherbrooke, Sherbrooke, QC Canada; 4 Department of Specialized Medicine, Division of Medical Genetics, McGill University Health Centre, Montreal, QC Canada; 5 Centre de Recherche de L’Institut Universitaire de Cardiologie et de Pneumologie de Québec (IUCPQ), Québec, QC Canada; 6 Department of Medicine, Faculty of Medicine, Université Laval, Québec, QC Canada; 7 Centre de Recherche du Centre Hospitalier Universitaire de Sherbrooke (CR-CHUS), Sherbrooke, QC Canada; 8 Department of Medicine, FMHS, Université de Sherbrooke, Sherbrooke, QC Canada; 9 Department of Population Medicine, Harvard Pilgrim Health Care Institute, Harvard Medical School, Boston, USA; 10 Diabetes Unit, Massachusetts General Hospital, Boston, USA

**Keywords:** microRNA, Next-generation sequencing, Maternal plasma, Circulating microRNA, Pregnancy

## Abstract

**Background:**

During pregnancy, maternal metabolism undergoes substantial changes to support the developing fetus. Such changes are finely regulated by different mechanisms carried out by effectors such as microRNAs (miRNAs). These small non-coding RNAs regulate numerous biological functions, mostly through post-transcriptional repression of gene expression. miRNAs are also secreted in circulation by numerous organs, such as the placenta. However, the complete plasmatic microtranscriptome of pregnant women has still not been fully described, although some miRNA clusters from the chromosome 14 (C14MC) and the chromosome 19 (C19MC and miR-371-3 cluster) have been proposed as being specific to pregnancy. Our aims were thus to describe the plasma microtranscriptome during the first trimester of pregnancy, by assessing the differences with non-pregnant women, and how it varies between the 4^th^ and the 16^th^ week of pregnancy.

**Methods:**

Plasmatic miRNAs from 436 pregnant (gestational week 4 to 16) and 15 non-pregnant women were quantified using Illumina HiSeq next-generation sequencing platform. Differentially abundant miRNAs were identified using DESeq2 package (FDR q-value ≤ 0.05) and their targeted biological pathways were assessed with DIANA-miRpath.

**Results:**

A total of 2101 miRNAs were detected, of which 191 were differentially abundant (fold change < 0.05 or > 2, FDR q-value ≤ 0.05) between pregnant and non-pregnant women. Of these, 100 miRNAs were less and 91 miRNAs were more abundant in pregnant women. Additionally, the abundance of 57 miRNAs varied according to gestational age at first trimester, of which 47 were positively and 10 were negatively associated with advancing gestational age. miRNAs from the C19MC were positively associated with both pregnancy and gestational age variation during the first trimester. Biological pathway analysis revealed that these 191 (pregnancy-specific) and 57 (gestational age markers) miRNAs targeted genes involved in fatty acid metabolism, ECM-receptor interaction and TGF-beta signaling pathways.

**Conclusion:**

We have identified circulating miRNAs specific to pregnancy and/or that varied with gestational age in first trimester. These miRNAs target biological pathways involved in lipid metabolism as well as placenta and embryo development, suggesting a contribution to the maternal metabolic adaptation to pregnancy and fetal growth.

**Supplementary Information:**

The online version contains supplementary material available at 10.1186/s12958-021-00883-1.

## Background

The metabolic adaptations to pregnancy are challenging for the expecting women while being essential for embryonic and fetal development. Studies support that microRNAs (miRNAs) regulate embryonic and fetal growth and development as well as maternal metabolic adaptations and maintenance of pregnancy [1, 2]. Nonetheless, their roles, expression and temporal variations in pregnancy remain overall poorly understood.

miRNAs are non-coding short RNA molecules (19-25 nucleotides long) regulating gene expression at the post-transcriptional level by interacting with their target messenger RNA (mRNA) sequences [3]. miRNAs regulate most biological processes and play crucial roles in cellular proliferation and communication as well as in embryonic development. Following transcription from the nuclear genome and processing in the nucleus, pre-miRNAs are exported to the cytoplasm for further processing and maturation [4]. Mature miRNAs can directly regulate mRNA stability and gene translation of their target genes (mRNA) within the cell. They can also be exported and reach distant organs through blood circulation (or other fluids) where they regulate post-transcriptional gene expression [3]. miRNAs are stable in biological fluids and were associated with physiological and pathological conditions and related outcomes such as gestational diabetes mellitus (GDM) [5, 6]. As such miRNAs contribute to pregnancy development by regulating important steps like trophoblast differentiation and migration, embryo implantation and maternal-fetal immune tolerance [7–9].

Previous studies have demonstrated that miRNAs from clusters on chromosome 14 (C14MC) and chromosome 19 (C19MC and miR-371-3) measured both in placenta tissue and plasma are associated with pregnancy and differentially expressed between placentas from normal and complicated pregnancies [10–12]. These miRNAs are contained in regions involved in embryonic development and the regulation of cellular differentiation. Cluster-specific miRNAs are also known to be expressed in a time-dependent manner during pregnancy. According to previous studies, C14MC miRNAs are predominantly expressed during the first trimester and their expression decreases as pregnancy progresses. On the opposite, C19MC cluster is more strongly expressed by trophoblast cells in the third trimester of pregnancy, while their abundance is lower in early pregnancy [10, 11]. Nonetheless, the plasmatic microtranscriptome of pregnant women has not yet been comprehensively investigated although this knowledge is key to better understanding physiological roles of miRNAs in pregnancy [6]. We thus hypothesize that the microtranscriptome from first trimester pregnant women would differ from non-pregnant women and vary within the first trimester of pregnancy. These pregnancy-associated miRNAs would target biological pathways essential to pregnancy such as embryonic and fetal growth and development, maternal metabolic adaptation and maintenance of pregnancy.

Our primary goal was to characterize the plasmatic microtranscriptome of pregnant women during the first trimester of pregnancy by 1) comparing their miRNAs profile with non-pregnant women; and 2) assessing miRNAs abundance variations between the 4^th^ and 16^th^ week of pregnancy. We have also identified the biological pathways potentially regulated by these miRNAs for our secondary goal. To achieve our goals, we have sequenced the plasmatic miRNAs of 451 pregnant and non-pregnant women.

## Methods

### Pregnant participants selection

Pregnant women selected for this study were recruited as part of the Genetics of Glucose regulation in Gestation and Growth (Gen3G) birth cohort which has been previously described [13]. Briefly, inclusion criteria were women in reproductive age (≥ 18 years old) not taking any medication influencing glycemia and with a singleton pregnancy. Women were excluded if they had diabetes at their first visit (between the 4^th^ to 16^th^ weeks of pregnancy). Women with polycystic ovarian syndrome and antiphospholipid syndrome were also excluded from the analysis. For this study, selection criteria also comprised plasma sample availability at the first visit, mother and offspring follow-up until 5-years post-partum as well as availability of genetic (Mother: Infinium MEGA^EX^ Array, Illumina; Offspring: whole genome sequencing) and epigenetic (EPIC Array, Illumina) data. Women with complications such as GDM (*n* = 53), PE (*n* = 19) or GHTN (*n* = 26), or both complications (GDM and PE or GHTN; *n* = 6) were included in this study for a total of 444 women of European descent. The ethics review board of *CIUSSS de l’Estrie-CHUS* approved the study and each participant provided informed written consent for their participation in the study.

### Non-pregnant participants selection

For comparison with non-pregnant women, we have selected 15 women from the UNISON cohort which was described in Desgagné *et al*. [14]. In this cohort, 18 couples (men and women) were recruited based on the following inclusion criteria: ≥18 and ≤ 65 years old, body mass index (BMI) ≥18 and ≤ 35 kg/m^2^, were non-smoking or had no endocrine or metabolic disorder such as diabetes or dyslipidemia. Serum human chorionic gonadotrophin (*B*-HCG), follicle-stimulating hormone (FSH) and luteinizing hormone (LH) levels were measured by immunoassays on DXI 800 analyzer (Beckman Coulter) while estradiol levels were quantified on the Architect model (Abbot Core Laboratory). Three women were excluded because of their hormonal profile suggestive of a pregnancy (*n* = 2) or a menopause status (*n* = 1). Written informed consent was obtained from all participants at the beginning of the study and this project was approved by the *CIUSSS du Saguenay–Lac-St-Jean* and IUCPQ Ethics Committees.

### RNA extraction

Total RNA was extracted with the mirVana PARIS kit and protocol (Thermo Fisher Scientific, catalog # AM1556), in a randomized order, from either 500 μl of plasma 1 h following a 50 g glucose challenge test (pregnant women) or from 1.5 mL of plasma (12 h fasted state; non-pregnant women) kept at −80 °C until extraction. Seventy-five μl of nuclease-free water was used to eluate total RNA. Total RNA was precipitated following the method described by Burgos et al. [15]. In brief, 35 μl of cold (4 °C) 7 M ammonium acetate solution (Thermo Fisher Scientific, catalog # 02002268) were added to 70 μl of total RNA. This was followed by the addition of 420 μl of chilled (−20 °C) absolute ethanol (Commercial Alcohols, ON, Canada; catalog # P006EAAN) and an overnight precipitation at −20 °C. The mix was then centrifuged at 16,000 g at 4 °C for 30 min. Two hundred μl of cold 80% ethanol was used to wash the RNA pellet, and this was followed by centrifugation at 16,000 g at 4 °C for 5 min. This step was done twice. It was then dried for 30 min at room temperature and 5 μl of nuclease-free water was added. The complete volume was used for library preparation.

### Library preparation

RNA samples were randomized before library preparation. An adapted version of the Truseq Small RNA Sample Prep kit (Illumina, BC, Canada; catalog # RS-200-0012) protocol was used to cope for low concentrated miRNA samples as in Burgos *et al*. [15]. Briefly, only half of the standard protocol volumes were used for ligation (3′ and 5′ ends) of RNA sample (5 μl), reverse transcription, barcoding (index 1-20: one index per sample) and PCR amplification (15 cycles) to preserve the ratio between RNA amount (which is lower in plasma samples) and the reagents. Purification and size-selection (145-160 bp) of the libraries were done by migration on a Novex polyacrylamide TBE Gel, 6% (Thermo Fisher Scientific, catalog # EC6265BOX) followed by elution in 300 μl of nuclease-free water and overnight incubation at 500 RPM at room temperature on an incubating microplate shaker (VWR, ON, Canada, catalog #12620-930). The libraries were then precipitated according to the manufacturer instructions (including incubation of the precipitation mix at −80 °C for 30 min) and were resuspended in 25 μl of 10 mM Tris-HCI pH 8.5 buffer.

### Library quality control

Either the Agilent 2100 Bioanalyzer and Agilent High Sensitivity DNA Kit (Agilent, Mississauga, ON, Canada; catalog # 5067-4626) or the Kapa Illumina GA with Revised Primers-SYBR Fast Universal kit (Kapa Biosystems) and a LabChip GX (PerkinElmer, catalog# CLS760672) instrument were used to assess the quality of each library (i.e., concentration, library length and primer dimers absence). Quality control, pooling and sequencing of the libraries were performed at the McGill University and Génome Québec Innovation Centre (Québec, Canada).

### miRNA sequencing

The samples were sequenced on either the HiSeq 2500 or HiSeq 4000 platforms. qPCR was used to quantify each library which were equimolarly pooled (HiSeq 2500: 7pM final molarity; 12 libraries with different indexes per lane; HiSeq 4000: 10pM final molarity; 20 libraries with different indexes per lane), denatured and clustered on single-read Illumina flow cells (catalog # GD-401-3001 and catalog GD-410-1001) according to the manufacturer’s standard protocol. The libraries were then sequenced on Illumina HiSeq 2500 or HiSeq 4000 sequencing platforms for 50 cycles, with 7 cycles indexing read. To ensure comparability and standardization, 12 samples were extracted twice and sequenced on both platforms. As expected, these samples showed high miRNA level correlations (Pearson correlation coefficient ≥ 0.94; Supplementary Fig. 1) and confirmed that our sequencing approach is accurate.

### Bioinformatics processing of the sequencing data

The extra-cellular RNA processing toolkit (exceRpt) pipeline version 4.6.3 was used to process miRNA sequencing data [16]. Briefly, exceRpt use FASTX-Toolkit to remove adapter sequences and low quality reads (Phred score < 20 for 80% or more of the read). High quality sequences were mapped to the human genome (GRCh37) and miRBase version 21 with STAR. As there was little rRNA contamination in our samples (average of 1.04 ± 0.90% of total mapped reads) based on metrics from STAR, the reads were not mapped to rRNA to gain speed during the analysis. A total of 8 outlier samples were excluded from the analyses after performing data visualization of the raw read counts (7 samples with fewer than 500,000 miRNAs reads and 1 sample with more than 25,000,000 miRNAs reads) (Supplementary Fig. 2).

### Statistical analysis

Nonparametric Mann-Whitney U were applied to assess differences in participants’ characteristics between pregnant (Gen3G cohort) and non-pregnant (UNISON cohort). The *DESeq2* package was used to identify pregnancy-associated miRNAs [17]. Briefly, all miRNAs detected (with 1 read in at least 1 sample) were included in the analysis. Default DESEq2 parameters were applied including adjustment for False Discovery Rate (FDR) set at q-value < 0.1: only miRNAs with a fold change < 0.05 or > 2 and q-value < 0.05 were considered significant for this analysis. The *collapseReplicates* function of DESeq2 package was used to combine read counts from samples ran in duplicate on both sequencing platforms (*n* = 12). The model was first adjusted for potential technical biases (sequencing run and lane) and is the model presented in the results section. Sensitivity analyses included further adjustments for age, BMI and glycemia to verify whether those covariates contribute to the model, as well as removing participants with pregnancy complications and those that were not sequenced on the HiSeq 4000 platform (model 1: sequencing run and lane, age and BMI; model 2: sequencing run and lane, age, BMI and glycemia; model 3: sequencing run and lane without participants with GDM, PE or GHTN; model 4: sequencing run and lane, including participants that were sequenced on the HiSeq 2500 platform only).

miRNA abundance variations in maternal plasma between the 4^th^ and the 16^th^ week of pregnancy were also assessed using *DESeq2* package, again using the default parameters. Our analysis was performed by entering gestational age (in weeks) as a continuous variable in the model, while also correcting for both sequencing runs and lanes. Thus, the fold changes signify the variation in miRNA abundance for an increase of 1 week of gestation. Sensitivity analyses including covariates such as maternal age, BMI and excluding any pregnancy complications were also performed to evaluate their contribution to the model but did not show significant differences with the main results. The Volcano plots were generated using the *EnhancedVolcano* package [18]. R version 4.0.2 in R studio version 1.3.1056 was used for statistical analysis [19].

### Biological pathway analysis and miRNA-mRNA interaction network

Biological pathway analysis was carried out using DIANA-miRpath v.3 with the Kyoto Encyclopedia of Genes and Genomes (KEGG) annotations [20, 21]. For the source of interactions, the DIANA database Tarbase v7.0 was selected as it only considers experimentally validated miRNA:mRNA interactions [22]. Default settings in miRpath were used (*p*-value threshold < 0.05 after correction for FDR) for the enrichment analysis and the pathway union parameter were used for merging results by applying the Fisher Exact test (Hypergeometric Distribution). Target genes of the fatty acid biosynthesis, fatty acid metabolism, TFG-beta signaling pathway and ECM receptor interaction pathways were identified with miRpath. Networks of miRNA-mRNA interaction were constructed with Cytoscape version 3.8.3 using miRNA-mRNA interactions from the Tarbase v7.0 strong evidence database using these genes and miRNAs associated with pregnancy or gestational age.

## Results

### Description of the cohort

Characteristics of the participants are shown in Table 1. Overall, pregnant women were younger (median age: 28 vs 30 years; *p* = 0.035) but had a similar body mass index (BMI; median BMI: 24.2 vs 23.6 kg/m^2^; *p* = 0.245) as compared to non-pregnant women. Blood samples were collected between the 4^th^ and the 16^th^ week of pregnancy (median gestational age: 9.19 weeks).

**Table 1:** Characteristics of participants

Characteristics	Pregnant women (Gen3G) *n* = 436		Non-pregnant women (UNISON) *n* = 15		*p*-value
	Median	Range	Median	Range	
Age (years)	28	18 – 47	30	23 – 49	0.035
BMI (kg/m^2^)	24.2	16.1 – 54.1	23.6	18.6 – 31.0	0.245
Glycemia (mmol/L)	5.4^a^	2.6 – 10.2^a^	4.9	4.3 – 5.7	NA
Gestational age at first visit (weeks)	9.19	4.1 – 16.3	NA		NA

Mann-Whitney U-test

Abbreviations: *BMI* Body mass index, *NA* Not applicable, *SD* Standard deviation

^a^*N* = 404

### miRNAs identified between pregnant and non-pregnant women

After data processing and quality control assessment, we obtained an average of 16.7 ± 6.7 and 8.5 ± 1.7 million reads for the pregnant and non-pregnant women cohorts respectively, with a reads mapping rate of ~ 90% onto the human genome using the exceRpt pipeline [16]. A total of 2101 miRNAs were detected (≥ 1 normalized read count in at least 1 sample), of which 1012 (~ 48%) being detected in both pregnant and non-pregnant women (only 1 specifically detected in the later cohort). This leaves 1088 miRNAs detected only in pregnant women, most of them being weakly expressed and detected in < 15% samples on average.

**Fig. 1:**
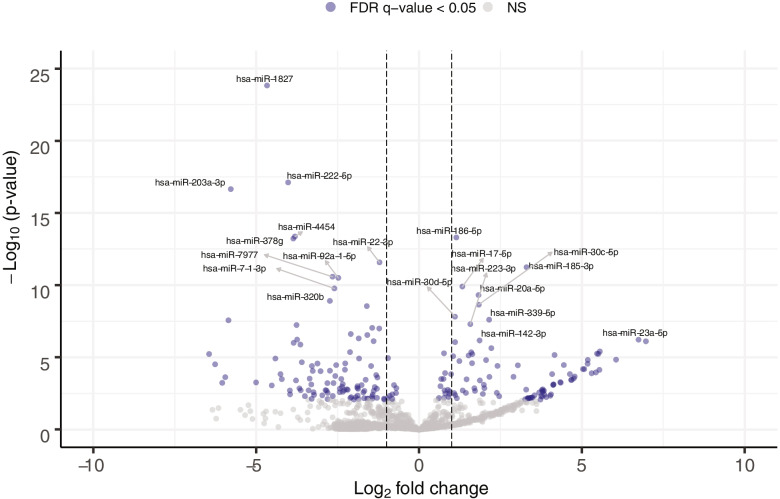
Plasmatic miRNAs associated with pregnancy. Vertical dotted lines represent an absolute Log2 fold change of 1 relatively to non-pregnant women. Blue circles represent miRNAs with an FDR adjusted q-value < 0.05. The top 20 most significantly associated miRNA with pregnancy (Supplementary Table 2) are labeled

We found 191 miRNAs that were differentially abundant (fold change < 0.05 and > 2, FDR q-value ≤ 0.05) between pregnant and non-pregnant women. These miRNAs are shown in Fig. 1 and a complete list is provided in Supplementary Table 1. Further statistical adjustment for age, BMI and glycemia as well as removal of complicated pregnancies (GDM, GHT, PE) did not significantly affect the results as nominal *p*-values and fold changes remain similar (data not shown). Interestingly, 52 (~ 27%) of the differentially abundant miRNAs have an important difference in the proportion of women in which the miRNAs were detected (> 50% in one group as compared to the other), when using a detection threshold of 1 normalized count (Supplementary Table 1). For example, hsa-miR-1283 has been detected in 98.4% of the samples collected in pregnancy whereas it was not detected in any of the 15 samples from non-pregnant women.

All miRNAs from the C14MC, C19MC and 371-3 clusters known to be pregnancy-associated were detected in at least one pregnant woman, and 92 were detected in at least 1 non-pregnant women. However, a fraction of the miRNAs from C14MC were identified as differentially abundant (7 more and 4 less) in pregnant women as compared to non-pregnant women (Supplementary Table 2). Thirteen miRNAs of the C19MC cluster that were differently abundant between cohorts were all more present in pregnant women, whereas the only differentially abundant miRNA (miR-371b-5p) we identified from the 371-3 cluster was more abundant in non-pregnant group (Supplementary Table 2).

In addition to miRNAs from the three clusters previously associated with pregnancy, 166 other miRNAs were also identified as differently abundant (Supplementary Table 1). Of these, 71 were more abundant in pregnant women whereas 95 were less abundant. Supplementary Table 3 displays the top 10 miRNAs that were found more and less abundant in pregnant versus non-pregnant women.

### Biological pathway analyses and interaction network of miRNAs associated with pregnancy

**Fig. 2:**
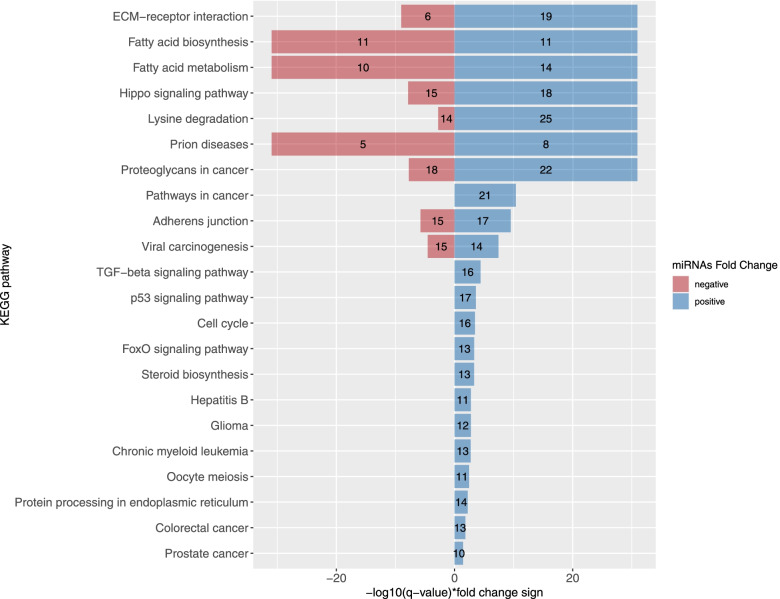
Pathway analysis of pregnancy-associated miRNAs. KEGG pathways most significantly enriched with genes targeted by the miRNAs associated with pregnancy are represented in relation with their respective FDR adjusted q-value. The number in the bars is the number of miRNAs targeting a pathway

**Fig. 3:**
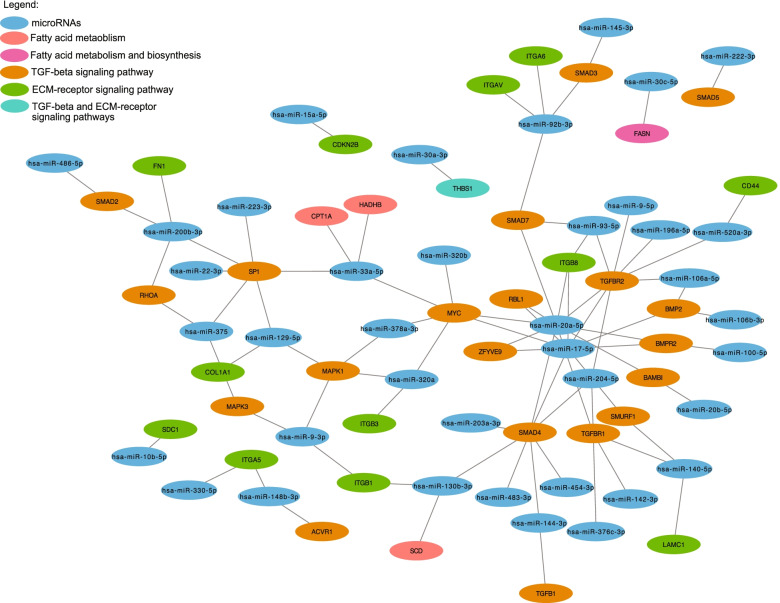
miRNA-mRNA interaction analysis of miRNAs associated with pregnancy. Colors represent miRNAs and different biological pathways

Figure 2 shows the metabolic pathways targeted by the 191 miRNAs identified as associated with pregnancy status. MiRNAs more and less abundant in pregnancy target 22 and 9 metabolic pathways, respectively. Interestingly, the extracellular matrix (ECM)-receptor interaction pathway and both fatty acid metabolism and biosynthesis pathways were common to both more and less abundant miRNAs, while the tumor growth factor beta (TGF-beta) signaling pathways was targeted only by more abundant miRNAs. The targeted genes by these miRNAs of fatty acid biosynthesis and metabolism pathways as well as ECM-receptor interaction and TGF-beta signaling pathways are shown in Fig. 3 (only Tarbase v.7 strong interactions were analyzed).

### miRNAs also vary according to gestational age during the first trimester of pregnancy

**Fig. 4:**
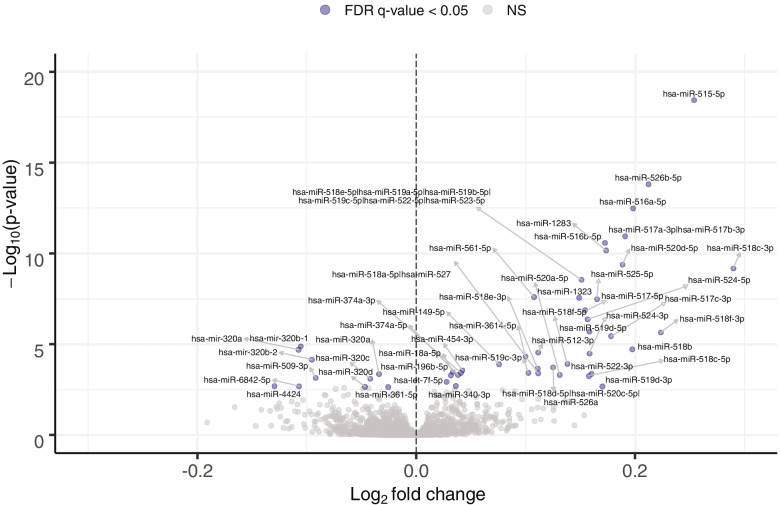
Plasmatic miRNAs associated with advancing gestational age during first trimester. The abundance of miRNAs represented by a blue circle are varying significantly during first trimester (FDR q-value ≤ 0.05). Fold changes represent the change in miRNA abundance for each increase of 1 week of gestation during first trimester of pregnancy

Regarding miRNA abundance variations between the 4^th^ and 16^th^ week of pregnancy, 57 unique miRNAs were significantly associated with advancing gestational age from 4 to 16 weeks of pregnancy (FDR q-value ≤ 0.05; Fig. 4). From these, 47 and 10 miRNAs were positively and negatively associated with advancing gestational age, respectively (Supplementary Fig. 3). The observed fold changes are relatively small (0.91 to 1.22) as they represent the variation in miRNA abundance for each increase of 1 week of gestation during first trimester of pregnancy. More than half of these miRNAs (30 miRNAs) were detected in at least 90% of pregnant women (Supplementary Table 4). Of the miRNAs that varied in abundance according to gestational week, 37 miRNAs were localized on C19MC and were all positively associated with increasing gestational age. The remaining 20 miRNAs were not located on any known pregnancy-associated clusters (Supplementary Table 4).

Interestingly, the abundance of 17 miRNAs associated with pregnancy status also varied with gestational age. Those miRNAs were: hsa-miR-320a, hsa-miR-1323, hsa-miR-512-3p, hsa-miR-516b-5p, hsa-miR-454-3p, hsa-miR-374a-5p, hsa-miR-1283, hsa-miR-518e-5p, hsa-miR-519a-5p, hsa-miR-519b-5p, hsa-miR-519c-5p, hsa-miR-522-5p, hsa-miR-523-5p, hsa-miR-320c, hsa-miR-374a-3p, hsa-miR-516a-5p, hsa-miR-526b-5p. As expected, most of them are part of the C19MC cluster (12 miRNAs) and were found to be more abundant in pregnant women and positively associated with gestational age.

### Biological pathway analyses of miRNAs associated with advancing gestational age

**Fig. 5:**
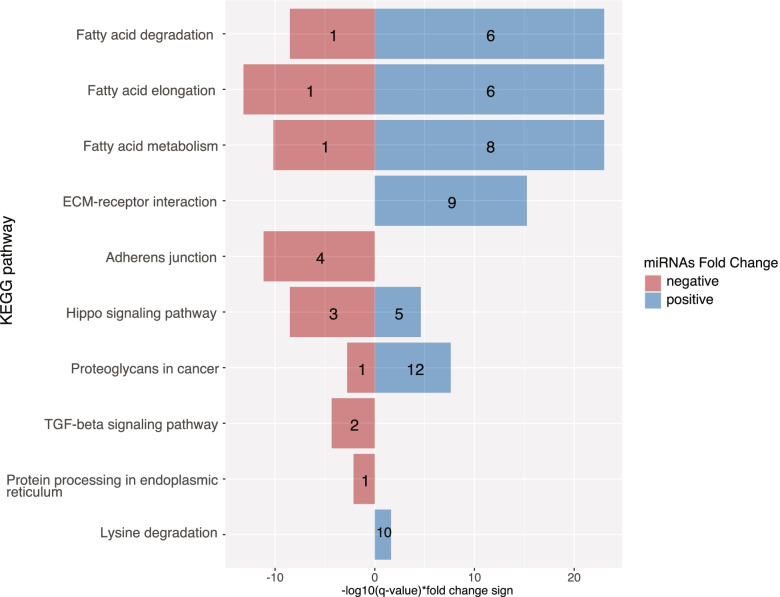
Biological pathway analysis of miRNAs associated with gestational age at first trimester. Histogram of KEGG biological pathways significantly targeted (FDR q value ≤ 0.05) by miRNAs found more or less abundant with increasing gestational age during first trimester based on experimentally validated miRNA:mRNA interactions (Tarbase v7.0). The numbers in the histogram bars represent the number miRNAs which targeted each pathway

**Fig. 6:**
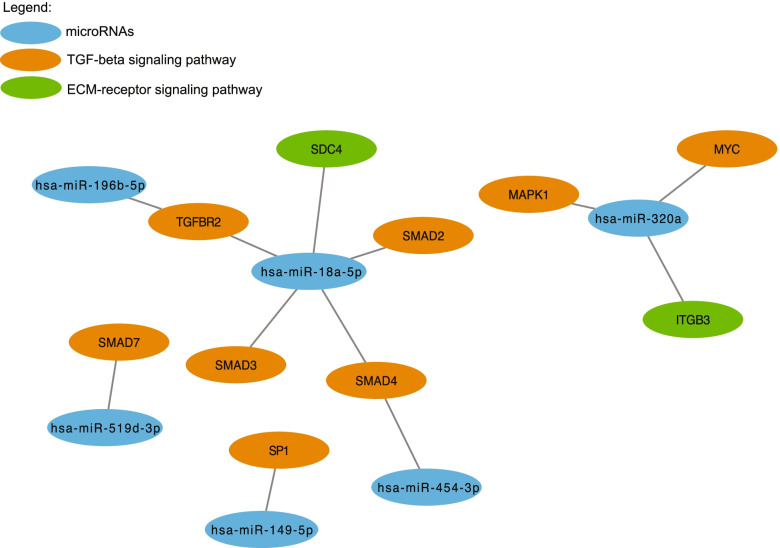
miRNA-mRNA interaction analysis of miRNAs associated with pregnancy. Colors represent miRNAs and different biological pathways

Biological pathway analysis of the 57 miRNAs that were associated with advancing gestational age revealed that 7 and 8 pathways were respectively targeted by the miRNAs positively and negatively associated greater gestational age (FDR q-value ≤ 0.05) (Fig. 5). Notably, pathways such as fatty acid degradation, fatty acid elongation and fatty acid metabolism were targeted by both more and less abundant miRNAs, while the ECM-receptor interaction and TGF-beta pathways were targeted by miRNAs that were positively and negatively associated with gestational age, respectively. A miRNA-mRNA interaction network (Fig. 6) shows the genes involved in ECM-receptor interaction and TGF-beta signaling pathways that are targeted by these miRNAs (only Tarbase v.7 strong interactions were analyzed).

## Discussion

Although few studies have reported miRNAs associated with pregnancy, these were mainly restricted to miRNAs from known pregnancy-associated clusters in the placenta, or were conducted using maternal plasma/serum in late pregnancy [6, 12, 23]. To our knowledge, this is the first study characterizing the whole maternal plasmatic microtranscriptome at first trimester of pregnancy, and identifying miRNAs associated with pregnancy and their temporal variation within the first weeks of pregnancy.

Among the 2101 miRNAs with at least 1 normalized count detected in plasma from at least one pregnant and non-pregnant women, we identified 191 miRNAs that were associated with pregnancy status and 57 unique miRNAs which circulating levels were associated with advancing gestational age between 4 to 16 weeks of pregnancy. As expected, two third of these miRNAs are located within the three known pregnancy-related miRNA clusters. Surprisingly, only 7 out of 11 differentially abundant miRNAs from the C14MC were more concentrated in the pregnant group, while the other 4 miRNAs were less abundant in pregnancy raising some questions about sources other than the placenta [11, 24–27]. Only one miRNA from the miR-371-3 cluster showed difference between groups being less abundant in pregnant women. To the best of our knowledge, it is the first time that miR-371-3 cluster was quantified in plasma. As such, these variations in plasma, or lack of, might not reflect previous finding in placenta tissue [10, 11, 28]. All miRNAs from the C19MC cluster were more abundant in the pregnant group and positively associated with gestational age, which is in line with previous studies [10–12].

The 17 miRNAs associated both with pregnancy status and advancing gestational age were mostly from the C19MC cluster, which has been previously found to be specific to pregnancy and expressed more abundantly as pregnancy progresses [10–12]. The other miRNAs common to both lists were not part of known pregnancy clusters nor were they previously associated with pregnancy and its progression, although some of them have been associated with pregnancy’s complications. Both hsa-miR-320a (less abundant in pregnancy and negatively associated with advancing gestational age) and hsa-miR-454-3p (higher in pregnant women and with advancing gestational age) have been associated with trophoblast invasion and PE. Meanwhile, hsa-miR-374a-5p which was both higher in pregnant women and with advancing gestational age has previously been associated with preterm delivery [29–31].

Biological pathway analysis of miRNAs associated with pregnancy and gestational age revealed 22 and 29 miRNAs regulate fatty acid biosynthesis and fatty acid metabolism pathway respectively. Among the targeted genes identified, *FASN* (fatty acid synthase) encodes the fatty acid synthetase enzyme which catalyzes the de novo biosynthesis of long-chain fatty acids [32]. The interaction network also revealed targets such as *HADHB* (hydroxyacyl-CoA dehydrogenase trifunctional multienzyme complex subunit B) and *SCD* (stearyl CoA desaturase). Both these genes encode for enzymes essentials for β-oxidation of long chain-fatty acids, which provide a significant source of energy [33]. Early in pregnancy, women are generally characterized by a slightly more insulin sensitive and lipogenic profile than later in pregnancy, allowing to store nutrients that will be later used to meet the metabolic demand of both the mother and her growing fetus during pregnancy, but also during the lactation period as well [34, 35]. By targeting these three genes, our pregnancy-associated miRNAs could play an early role in the regulation of the lipid metabolism pathways which is an important maternal metabolic adaptation needed to ensure sufficient nutrient supply to the mother and the growing fetus [36, 37].

Interestingly, 30 unique miRNAs both more abundant with pregnancy as well as positively associated with gestational age during first trimester target genes encoding for components of the ECM-receptor interaction pathway such as *ITGA5, ITGA6, ITGAV, ITGB1, ITGB3, ITGB8* (integrin subunits alpha 5, 6, V and beta 1, 3, 8), *LAMC1* (laminin subunit gamma 1*), FN1* (fibronectin) and *COL1A1* (collagen type 1 subunit alpha 1). ECM remodeling of the myometrium occurs throughout pregnancy, promoting placentation, embryo implantation and cell differentiation and later adapting to the developing fetus [38]. This remodeling involves dynamic variation in the expression of laminin, fibronectin and collagen proteins [39–41]. Regulation of these biological processes by the identified miRNAs support their involvement in establishment of pregnancy and its maintenance. Another relevant pathway identified by our analysis is the TGF-beta signaling pathway. Indeed, a total of 18 miRNAs target different genes of this signaling pathway, such as the *SMAD* family proteins and *TGFBR* (TGF-beta receptors) 1 and 2. While having numerous physiological roles, evidence suggested that TGF-beta pathway plays an active role in a successful pregnancy by promoting embryo implantation and differentiation and placenta development during the first trimester [42–44].

## Strenghts and limitations

To the best of our knowledge, no other study identified miRNAs associated with pregnancy or varying during the first trimester of pregnancy. Importantly, we were able to find miRNAs that have not been previously linked to pregnancy by quantifying them with next generation sequencing, a technology both sensitive and allowing a comprehensive profiling of all miRNAs [45]. Our study included a large number of pregnant women carefully followed up throughout pregnancy, which allowed to adjust our analyses for possible confounders such as maternal age. Moreover, we validated that women from the UNISON cohort were not pregnant nor in menopause with hormonal dosages (data not shown) [14]. One limitation of our study is the relatively small number of non-pregnant women as well as their relatively lower sequencing reading depth as compared to those obtained with the samples from pregnant women. The results should thus be interpreted with caution, particularly for the miRNAs that were detected only in a small number of non-pregnant women and that were less abundant. However, a sensitivity analysis that included only pregnant (*n* = 56) and non-pregnant (*n* = 15) women for which miRNAs sequencing depth were similar demonstrated that most of the reported associations with pregnancy remained (data not shown). Additional analyses of different subsets of randomly selected pregnant women (*n* = 15) with similar sequencing depth as the 15 non-pregnant women revealed that more miRNAs were detected in each subset of the pregnant women compared to the non-pregnant women. Moreover, we did compute achieved statistical power (post-hoc power) for each of the 186 miRNAs we found associated with pregnancy. We based this analysis on a t-test (we used DESeq2 for our differential analysis) and pooled standard deviation (SD) which is a straightforward and appropriate approach in this context. Using a *p*-value of 0.0086 (corresponding to the FDR q-value of 0.05) as threshold, we found that 147 out of 186 differentially abundant microRNAs (79%) achieved an observed power ≥ 80% (Supplementary Table 5). Although we might have missed differences of small effect size, this analysis shows that our statistical power is appropriate (≥ 80%) for most of the miRNAs we have identified and that our study design is overall robust. It is also important to note that while the pregnant women had their blood drawn 1 h post glucose challenges, samples from the non-pregnant cohort were obtained after a 12 h fast. However, a sensitivity analysis including age, BMI and glycemia as a covariate did not change significantly the current results of this study (Supplementary Table 6). Another limitation is our incapacity to replicate our results in independent cohorts. Indeed, we have been unsuccessful to find data from technically comparable analysis and a similar cohort of non-pregnant women both in public databases such as ExRNA atlas and collaborators [46]. Also, the role of miRNAs in pregnancy was evaluated with pathway analyses and should be validated with functional studies, even if we only used mRNA and miRNA associations that have been validated experimentally (Tarbase v7.0 database).

## Conclusion

We identified circulating miRNAs that were associated with pregnancy status and which circulating levels are associated with advancing gestational age in early pregnancy. These miRNAs target genes in biological pathways that could be involved in maternal metabolic adaptations to pregnancy as well as embryonic and fetal implantation and development. Those miRNAs could be involved in the pathophysiology of the complications of pregnancy and potentially used as biomarkers of such conditions, although further studies are needed to confirm this hypothesis.

## Supplementary Information

**Additional file 1: Supplementary Figure 1.** miRNA abundance correlation between replicates from samples sequenced on two sequencing platforms. Each dot represents a unique miRNA log_10_ normalized read count from the HiSeq 2500 (replicate A) or the HiSeq 4000 platform (replicate B).

**Additional file 2: Supplementary Figure 2.** QQ-plot of the total number of reads mapped on miRNAs per sample. Each point represents a sample. Pregnant (in blue) and non-pregnant women (in green) samples were similar in terms of number of miRNAs total raw read counts as seen by the overlap of the data.

**Additional file 3: Supplementary Figure 3.** Spaghetti plot of the miRNAs associated with pregnancy. The relative abundance of miRNAs positively (A) or negatively (B) associated was obtained by dividing the normalized count of a given miRNA in a sample by the highest normalized count observed for this miRNA in all samples. For visualization purpose, gestational age is represented in intervals of pregnancy weeks. The means and the standard error of mean of the relative abundance for each week interval are displayed. The miRNAs are ranked according to their abundance (mean normalized count and detection rate in samples).

**Additional file 4. Supplementary Table 1.** Complete list of miRNAs differentially abundant in plasma from pregnant women at first trimester compared to non-pregnant women.

**Additional file 5. Supplementary Table 2.** miRNAs differentially abundant in plasma from pregnant women at first trimester compared to that of non-pregnant women and localized within the C14MC, C19MC and 371-3 clusters.

**Additional file 6. Supplementary Table 3.** Top 10 miRNAs differentially abundant in plasma from pregnant women at first trimester compared to that of non-pregnant women that are not encoded in known pregnancy-related miRNAs clusters.

**Additional file 7. Supplementary Table 4.** Variation in plasmatic miRNAs abundance during first trimester of pregnancy.

**Additional file 8. Supplementary Table 5.** Achieved statistical power (post-hoc power) for miRNAs associated with pregnancy.

**Additional file 9. Supplementary Table 6.** miRNAs associated with pregnancy analysis corrected for age, BMI and glycemia as well as run and sequencing lane.

## Data Availability

The datasets used and/or analyzed during the current study are available from the corresponding author upon reasonable request.

## Abbreviations

*B*-HCG: Human chorionic gonadotrophin
BMI: Body mass index
C14MC: Chromosome 14 microRNA cluster
C19MC: Chromosome 19 microRNA cluster
ExceRpt: Extra-cellular RNA processing toolkit
FDR: False Discovery Rate
FSH: Follicle-stimulating hormone
GDM: Gestational diabetes mellitus
GHTN: Gestational hypertension
LH: Luteinizing hormone
PE: Preeclampsia
*HADHA*: *Hydroxyacyl-CoA dehydrogenase trifunctional multienzyme complex subunit alpha*
*HADHB*: *Hydroxyacyl-CoA dehydrogenase trifunctional multienzyme complex subunit beta*
miRNA: microRNA
