# Supplementary figures and images for: Human plasma pregnancy-associated miRNAs and their temporal variation within the first trimester of pregnancy
Source: Reprod Biol Endocrinol. 2022 Jan 14;20:14. doi: 10.1186/s12958-021-00883-1 (PMC8759232; doi:10.1186/s12958-021-00883-1)

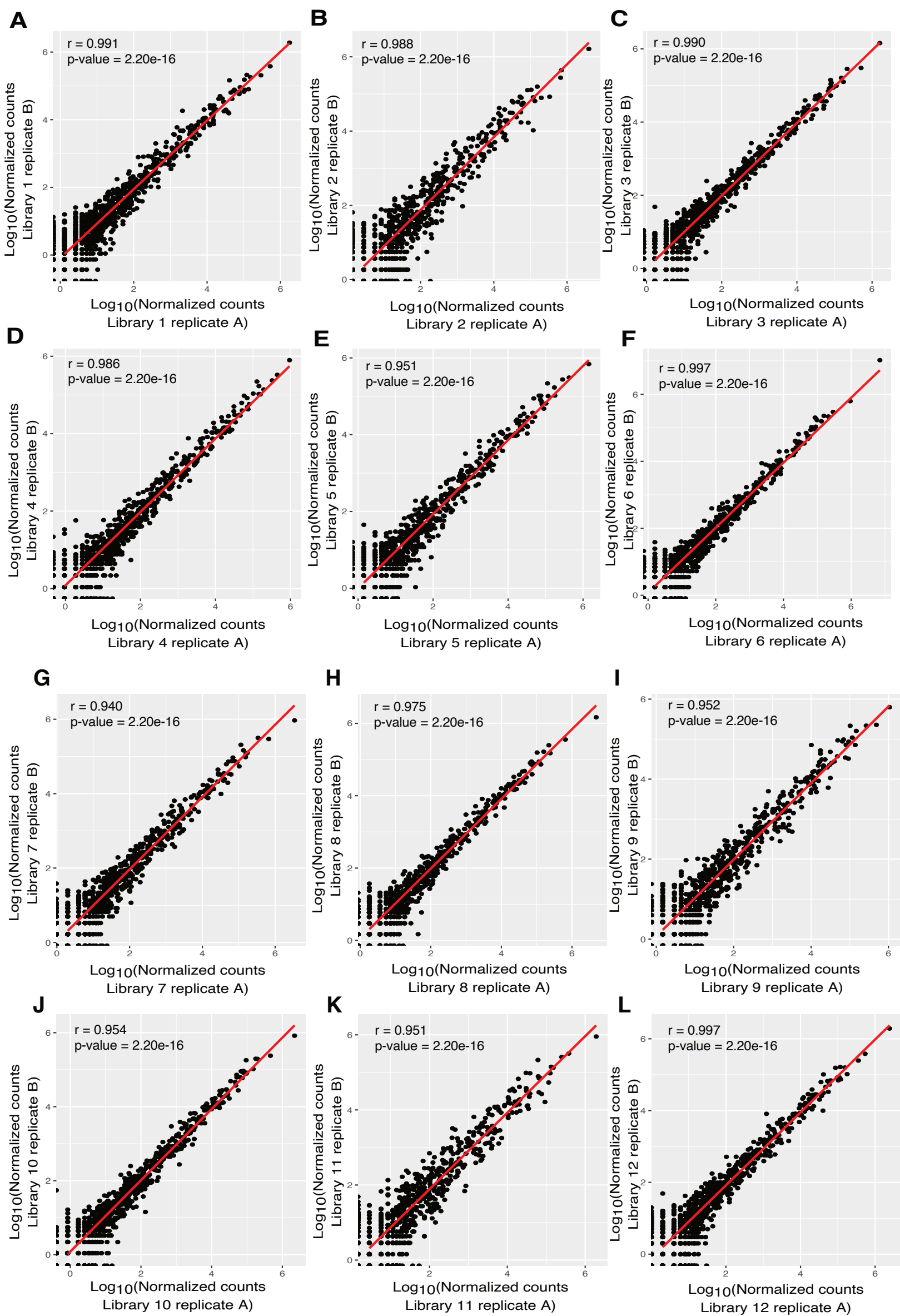

Supplement: Supplementary file 1 — Additional file 1: Supplementary Figure 1. miRNA abundance correlation between replicates from samples sequenced on two sequencing platforms. Each dot represents a unique miRNA log10 normalized read count from the HiSeq 2500 (replicate A) or the HiSeq 4000 platform (replicate B). [file 12958_2021_883_MOESM1_ESM.pdf]

Number of miRNAs total raw read counts per sample

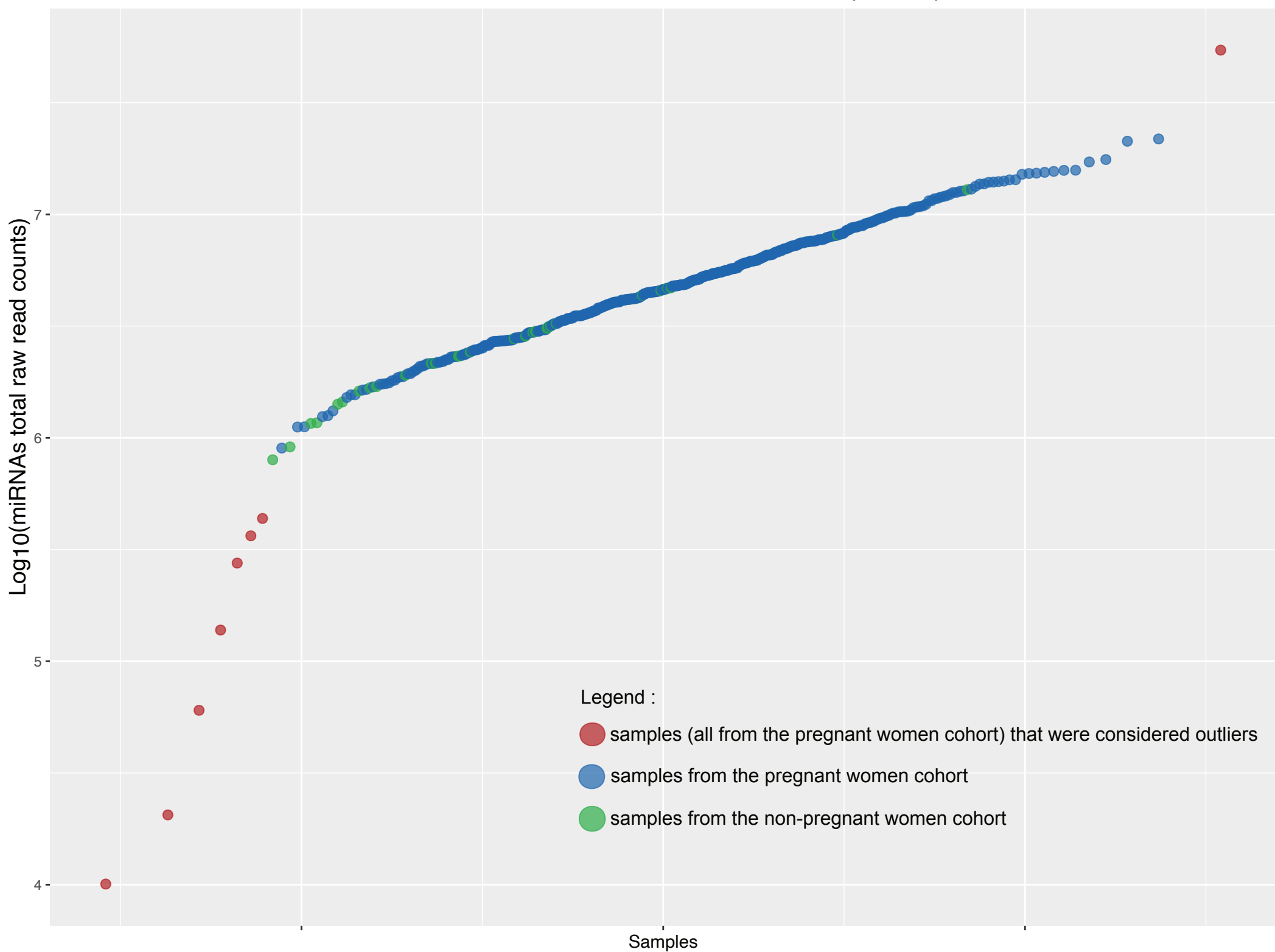

Supplement: Supplementary file 2 — Additional file 2: Supplementary Figure 2. QQ-plot of the total number of reads mapped on miRNAs per sample. Each point represents a sample. Pregnant (in blue) and non-pregnant women (in green) samples were similar in terms of number of miRNAs total raw read counts as seen by the overlap of the data. [file 12958_2021_883_MOESM2_ESM.pdf]

A

Relative abundance of miRNAs

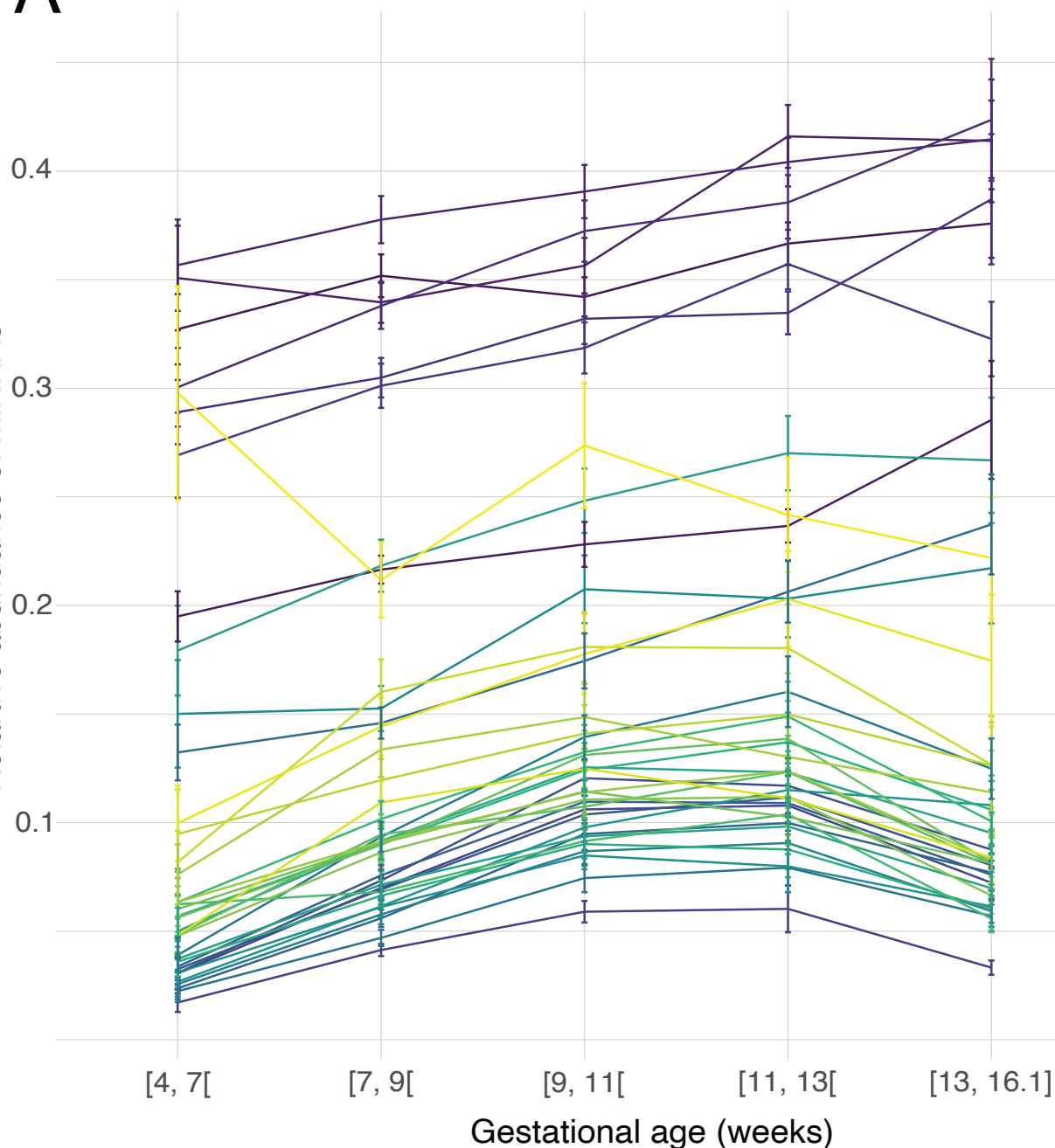

B

Relative abundance of miRNAs

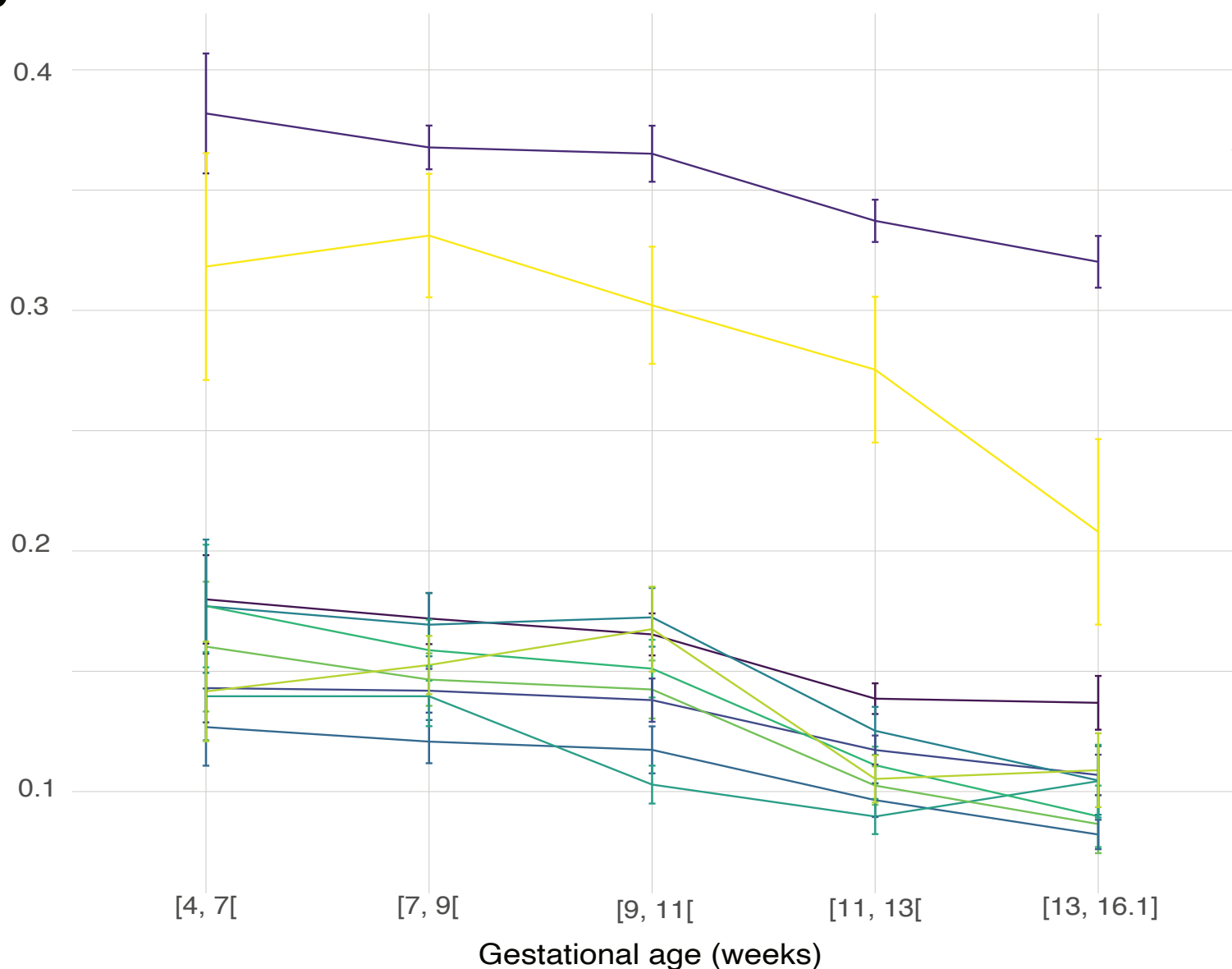

Supplement: Supplementary file 3 — Additional file 3: Supplementary Figure 3. Spaghetti plot of the miRNAs associated with pregnancy. The relative abundance of miRNAs positively (A) or negatively (B) associated was obtained by dividing the normalized count of a given miRNA in a sample by the highest normalized count observed for this miRNA in all samples. For visualization purpose, gestational age is represented in intervals of pregnancy weeks. The means and the standard error of mean of the relative abundance for each week interval are displayed. The miRNAs are ranked according to their abundance (mean normalized count and detection rate in samples). [file 12958_2021_883_MOESM3_ESM.pdf]
